# Arterial embolization in patients with grade-4 blunt renal trauma: evaluation of the glomerular filtration rates by dynamic scintigraphy with 99mTechnetium-diethylene triamine pentacetic acid

**DOI:** 10.1186/1757-7241-18-11

**Published:** 2010-03-07

**Authors:** Seiji Morita, Sadaki Inokuchi, Tomoatsu Tsuji, Tomokazu Fukushima, Shigeo Higami, Takeshi Yamagiwa, Iizuka Shinichi

**Affiliations:** 1 Department of Emergency and Critical Care Medicine, Tokai University School of Medicine, 143 Shimokasuya Isehara-City, Japan

## Abstract

**Background:**

High-grade blunt renal trauma has been treated by arterial embolization (AE). However, it is unknown whether AE preserves renal function, because conventional renal function tests reflect total renal function and not the function of the injured kidney alone. Dynamic scintigraphy can assess differential renal function.

**Methods:**

We performed AE in 17 patients with grade-4 blunt renal trauma and determined their serum creatinine (sCr) level and glomerular filtration rate (GFR; estimated by dynamic scintigraphy) after 3 months. In 4 patients with low GFR of the injured kidney (<20 ml·min^-1^·1.73 m^-2^), the GFR and sCr were measured again at 6 months. Data are presented as median and interquartile range (25th, 75th percentile).

**Results:**

The median GFR of the injured kidney, total GFR, and median sCr at 3 months were 29.3 (23.7, 35.3) and 96.8 (79.1, 102.6) ml·min^-1^·1.73 m^-2 ^and 0.6 (0.5, 0.7) mg/dl, respectively. In the patients with low GFR (ml·min^-1^·1.73 m^-2^), the median GFR of the injured kidney, total GFR, and median sCr (mg/dl) were 16.2 (15.7, 16.3), 68.7 (61.1, 71.6), and 0.7 (0.7, 0.9), respectively, at 3 months and 34.5 (29.2, 37.0), 90.9 (79.1, 98.8), and 0.7 (0.7, 0.8), respectively, at 6 months.

**Conclusions:**

The function of the injured kidney was preserved in all patients, indicating the efficacy of AE for the treatment of grade-4 blunt renal trauma.

## Background

Some recent studies have suggested that high-grade renal trauma can be successfully treated by non-operative management (NOM), which includes conservative management and arterial embolization (AE) [1-4]. In these studies, it was emphasized that NOM for high-grade renal trauma is less invasive than nephrectomy, and unlike nephrectomy, it preserves the renal function of the injured kidney. In most of these studies, renal function was assessed on the basis of the serum creatinine (sCr) level; serum blood urea nitrogen (BUN) level; and creatinine clearance (CCr_24 h_), which was determined from a 24-h urine sample. These parameters do not reflect the function of the injured kidney, but the total renal function (i.e., the function of both the injured and the contralateral uninjured kidney). Dynamic scintigraphy can determine the differential renal function.

We hypothesized that AE for severe blunt renal trauma could preserve the renal function of the injured kidney. Therefore, we used dynamic scintigraphy with ^99 m^technetium (Tc)-labeled diethylene triamine pentaacetic acid (DTPA) to evaluate renal function in patients with grade-4 blunt renal trauma (American Association for the Surgery of Trauma; AAST [5] after they had undergone AE.

## Methods

Between April 2003 and March 2008, we treated 28 patients with grade-4 blunt renal trauma (AAST) in Tokai University School of Medicine Emergency Center. Of these 28 patients, 6 underwent conservative management because extravasation of the contrast medium was not observed on computed tomography (CT), 21 underwent angiography and AE because extravasation of the contrast medium was observed on CT and angiography, and 1 underwent emergency nephrectomy because hemodynamic instability was present. In 17 of the 21 patients who underwent AE, the glomerular filtration rate (GFR) of the injured kidney was evaluated by dynamic scintigraphy at 3 months after the injury. Dynamic scintigraphy could not be performed in the remaining 4 patients because 3 of them died from multiple trauma and 1 patient refused treatment. In 4 of the 17 patients who underwent dynamic scintigraphy, the GFR of the injured kidney was less than 20 ml·min^-1^·1.73 m^-2^. In these patients, dynamic scintigraphy was repeated at 6 months after the injury.

Our case series included the 17 patients with grade-4 blunt renal trauma who underwent AE and whose renal function was evaluated by dynamic scintigraphy with ^99 m^Tc-DTPA at 3 months after the injury. In this case series, we report on detailed characteristics of these patients and examine whether renal function can be preserved by performing AE. Renal function was assessed on the basis of the GFR of the injured kidney, the contralateral uninjured kidney, and both kidneys (as estimated by dynamic scintigraphy) and sCr. In the case of the 4 patients who underwent dynamic scintigraphy at 3 and 6 months after the injury, we compared their GFR and sCr levels at these 2 time points. Data are presented as median and interquartile range (25th, 75th percentile).

In our institution, blunt abdominal trauma patients who are hemodynamically stable, with or without fluid resuscitation, undergo abdominal CT. If CT reveals grade-4 renal trauma with extravasation of the contrast medium, we perform emergency angiography. If angiography reveals extravasation of the contrast medium from the kidney, selective embolization or super-selective embolization is performed using a microcatheter and either gelatin particles or steel coils or both.

This study was approved by our hospital's Institutional Ethics Committee.

## Results

The detailed patient characteristics are presented in Table 1. Of the 17 patients, 14 were male; 9 patients were involved in a traffic accident, 4 sustained an injury during fall, 2 were victims of violence, and 2 sustained sports injuries. The median age of the patients was 35 (23, 41) years. The left kidney was injured in 10 patients; 1 patient had renal dysfunction due to diabetes mellitus, while the other patients had no relevant medical history. The median injury severity score (ISS) was 24 (16, 29). Ten patients had multiple trauma. AE was performed with gelatin particles (10 patients) or steel coils (3 patients) or both (4 patients). All patients survived and none experienced a recurrence of renal bleeding.

**Table 1:** Characteristics of the 17 patients

Patient's number	Sex	Age (years old)	Injured kidney	Cause of injury	Medical past history	ISS	Other major injuries	Embolization technique and materials
1.	F	23	L	T/A	-	29	Thoracic injury, Facial injury	SSE GP
2.	M	25	L	Fall	-	24	Thoracic injury, Limb Fx	SSE GP
3.	M	26	L	T/A	-	24	Thoracic injury, Limb Fx	SSE GP+SC
4.	F	45	L	T/A	-	29	Thoracic injury, Limb Fx	SE SC
5.	M	18	R	Sports	-	16	-	SSE GP
6.	M	52	R	Sports	DM	16	-	SSE GP
7.	M	37	R	T/A	-	24	Head injury, thoracic injury	SE SC
8.	M	36	R	Violence	-	16	-	SSE GP
9	M	16	R	Fall	-	36	Head injury, Pelvic Fx	SSE GP+SC
10.	M	25	L	T/A	-	16	-	SSE GP
11.	M	52	L	Fall	-	34	Thoracic injury, Pelvic Fx	SSE GP
12.	M	19	L	T/A	-	16	-	SSE GP+SC
13.	M	41	L	T/A	-	16	-	SE GP+SC
14.	M	35	R	Fall	-	24	Pelvic Fx, Limb Fx	SSE GP
15.	M	52	L	T/A	-	24	Head injury, Thoracic injury	SE SC
16.	M	38	R	T/A	-	16	-	SSE GP
17.	F	23	L	Violence	-	29	Head injury, Thoracic injury	SSE GP

F: female, M: male, L: left, R: right, T/A: traffic accident, DM: diabetes mellitus, Fx: fracture, SE: selective embolization,

SSE: super-selective embolization, GP: gelatin particles, SC: steel coils

The renal function at 3 months resented in Table 2. The median GFRs of the injured kidney, the contralateral uninjured kidney, and both kidneys at 3 months were 29.3 (23.7, 35.3), 59.4 (54.5, 73.9), and 96.8 (79.1, 102.6) ml·min^-1^·1.73 m^-2^, respectively. The median sCr was 0.6 (0.5, 0.7) mg/dl. In patients 3, 4, 6, and 10 (as listed in Table 1), the GFR of the injured kidney was less than 20 ml·min^-1^·1.73 m^-2 ^at 3 months. The GFR and sCr levels of these 4 patients at 3 and 6 months are presented in Table 3. Of these 4 patients, 3 were male; their median age was 35.5 (25.8, 46.8) years. For these 4 patients, the median GFRs of the injured kidney, contralateral uninjured kidney, and both kidneys at 3 months were 16.2 (15.7, 16.3), 53.0 (45.8, 55.3), and 68.7 (61.1, 71.6) ml·min^-1^·1.73 m^-2^, respectively, and the median sCr level was 0.7 (0.7, 0.9) mg/dl. For these 4 patients at 6 months, the median GFRs of the injured kidney, contralateral uninjured kidney, and both kidneys were 34.5 (29.2, 37.0), 55.5 (45.4, 65.4), and 90.9 (79.1, 98.8) ml·min^-1^·1.73 m^-2^, respectively, and the median sCr level was 0.7 (0.7, 0.8) mg/dl. The GFRs of the injured kidney and both kidneys improved.

**Table 2:** Renal function of the 17 patients at 3 months

	GFR at 3 months (ml·min^-1^·1.73 m^-2^)			
		
Patient's number	Injured kidney	Uninjured kidney	Both kidneys	sCr at 3 months (mg/dl)
1.	29.3	49.8	79.1	0.4
2.	39.2	59.4	98.6	0.6
3.	14.9	51.5	66.4	0.7
4.	16.0	57.7	73.6	0.7
5.	36.0	60.8	96.8	0.9
6.	16.3	28.8	45.1	1.6
7.	37.1	46.3	83.4	0.5
8.	26.4	76.2	102.6	0.6
9.	33.0	55.1	88.1	0.5
10.	16.4	54.5	70.9	0.5
11.	35.3	63.7	99.0	0.7
12.	28.6	74.2	102.8	0.4
13.	37.5	67.8	105.3	0.6
14.	23.7	89.6	113.3	0.5
15.	27.6	73.9	101.5	0.8
16.	34.2	81.4	115.6	0.7
17.	30.2	57.5	87.7	0.6

**Table 3:** Glomerular filtration rates at 3 and 6 months

	**GFR at 3 months (ml·min**^-1^**·1.73 m**^**-2**^**)**				**GFR at 6 months (ml·min**^**-1**^**·1.73 m**^**-2**^**)**			
				
Patient's number	Injured kidney	Uninjured kidney	Both kidneys	sCr at 3 months (mg/dl)	Injured kidney	Uninjured kidney	Both kidneys	sCr at 6 months (mg/dl)
3.	14.9	51.5	66.4	0.7	38.4	50.5	88.9	0.7
4.	16.0	57.7	73.6	0.7	32.4	60.4	92.8	0.7
6.	16.3	28.8	45.1	1.6	19.4	30.2	49.6	1.0
10.	16.4	54.5	70.9	0.5	36.5	80.2	116.7	0.5

## Discussion

Conservative management has become the standard treatment for patients with blunt renal trauma (AAST grades 1 to 3) who are hemodynamically stable [1-4]. Most experts agree that surgical exploration is required in patients with grade-5 blunt renal trauma. The management of patients with grade-4 blunt renal trauma, however, remains controversial [6-8]. Although ideally the surgical management of patients with severe blunt renal trauma should entail renal reconstruction, nephrectomy is required in majority of such patients. Hemodynamic instability in patients with blunt renal trauma is the most likely indication for nephrectomy, which is the most expeditious surgical option in this scenario. It is reported that nephrectomy is performed in 43-75% of patients who undergo emergency laparotomy for severe blunt renal injury [9,10]. Nephrectomy is the intentional removal of a kidney and necessarily results in partial loss of renal function. Therefore, unless nephrectomy is absolutely indicated, it constitutes an unacceptable infliction of iatrogenic injury.

In many recent studies, high success rates have been obtained with NOM, which includes conservative management and AE, of patients with high-grade blunt renal trauma [1-4]. NOM is therefore gradually becoming the recommended clinical treatment for high-grade blunt renal trauma, particularly in the case of hemodynamically stable patients. Although it is known that conservative management of patients with high-grade blunt renal trauma allows the injured kidney to be preserved and obviates the need for nephrectomy, it has remained unclear whether conservative management preserves the function of the injured kidney. This is because most previous studies have assessed renal function after NOM on the basis of the sCr and BUN levels and CCr_24 h _[1-4]. Levels of sCr and BUN are poor indicators of the function of the injured kidney, because the contralateral uninjured kidney can maintain normal serum concentrations of these markers. CCr_24 h _reflects the total renal function and not the function of the injured kidney alone. We consider radionuclide scanning to be a suitable examination for directly evaluating the function of the injured kidney, because it is the only examination that can assess differential renal function.

A few studies have used dynamic scintigraphy with ^99 m^Tc-dimercaptosuccinic acid (DMSA) for the morphological evaluation of the injured kidney [11-13]. By performing radionuclide renography and scintigraphy, Wessells et al. quantified the degree of preservation of renal function after reconstruction for traumatic renal injury (grades 2-5) [11]. They used ^99 m^Tc-DMSA and evaluated the function of the injured kidney on the basis of the uptake percentage. They defined adequate renal preservation as the salvage of more than one third of the injured kidney and reported that adequate preservation was achieved in 81% of their patients. By performing ^99 m^Tc-DMSA scintigraphy and CT angiography, El-Sherbiny et al. evaluated renal function and morphology long after conservative management in children with severe renal trauma [12]. They found no significant functional loss in any of the affected kidneys (split renal function, 41-50%).

Recent advances in radiological techniques such as CT and echography now allow these techniques to be used for the morphological evaluation of renal trauma patients; therefore, ^99 m^Tc-DMSA scintigraphy is not frequently used for this purpose. Compared to dynamic studies with ^99 m^Tc-DMSA, those with agents such as ^99 m^Tc-diethylenetriamine pentaacetic acid (DTPA), ^131^I- and ^123^I-ortho-iodohippurate (OIH), and ^99 m^Tc-mercaptoacetyl-glycyl-glycyl-glycine (MAG_3_) provide more information about differential renal function; in addition to GFR, the effective renal plasma flow (ERPF) can be calculated as a differential renal function.

In our case series, the median GFR of the injured kidney and the median sCr level at 3 months after the injury were 29.3 (23.7, 35.3) ml·min^-1^·1.73 m^-2 ^and 0.6 (0.5, 0.7) mg/dl, respectively. Further, the median GFR of both kidneys at 3 months was 96.8 (79.1, 102.6) ml·min^-1^·1.73 m^-2^. We therefore believe that adequate preservation of the function of the injured kidney was achieved. In the 4 patients in whom the GFR of the injured kidney was less than 20 ml·min^-1^·1.73 m^-2^, the median GFRs of the injured kidney and both kidneys at 3 months were 16.2 (15.7, 16.3) and 68.7 (61.1, 71.6) ml·min^-1^·1.73 m^-2^, respectively. This shows that adequate preservation of renal function was not achieved at 3 months. However, at 6 months, the GFRs of the injured kidney and both kidneys improved and were 34.5 (29.2, 37.0) and 90.9 (79.1, 98.8) ml·min^-1^·1.73 m^-2^, respectively. The GFR of both kidneys at 6 months was almost in the normal range. In patient 6, who had diabetic nephropathy before injury, the GFRs at 3 and 6 months did not show improvement. This suggests that blunt renal trauma patients with preexisting chronic kidney diseases may require careful long-term follow-up after AE. Furthermore, Wessells et al. reported that blunt renal trauma patients who develop hypotension in their clinical course experience significant renal dysfunction [11].

## Conclusions and Limitation

In our case series, AE in grade-4 blunt renal trauma patients resulted in the adequate preservation of renal function at 3 or 6 months after injury. This outcome suggests that AE is efficacious for the treatment of patients with grade-4 blunt renal trauma. However, because our research was a case series (n = 17), it does not provide enough evidence to prove this association. Further research, with a large number of patients should be conducted in future to examine this concept in more depth.

## Competing interests

The authors declare that they have no competing interests.

## Authors' contributions

SM conceived of this study, performed the analysis and prepared the manuscript.

TT, TF, SH, TY, IS contributed to the study design and prepared the figures.

SI participated as expert instructors, contributed to the study design.

All authors read and approved the final manuscript.
